# Re: Effects of serial macrocyclic-based contrast materials gadoterate meglumine and gadobutrol administrations on gadolinium-related dentate nuclei signal increases in unenhanced T1-weighted brain: a retrospective study in 158 multiple sclerosis (MS) patients

**DOI:** 10.1007/s11547-018-0864-9

**Published:** 2018-02-23

**Authors:** Jean-Sébastien Raynaud, Elisabeth Darmon-Kern, Eric Lancelot, Anne-Coline Laurent, Pierre Desché

**Affiliations:** Guerbet, BP 57400, 95943 Roissy CDG Cedex, France

In the September 2017 on‐line issue of La Radiologia Medica, Splendiani et al. published a clinical study on the effects of multiple injections of the macrocyclic GBCAs gadoterate meglumine and gadobutrol on dentate nuclei T1-weighted signal intensities (SI) in patients with multiple sclerosis [1]. In the abstract, the authors concluded: “SI increases on unenhanced T1-weighted images possibly indicative of gadolinium retention occur after serial administrations of the macrocyclic GBCAs, gadoterate meglumine and gadobutrol”.

This conclusion is, however, not supported by the results presented in the core of the article and the abstract.

The authors showed an increase in dentate nucleus-to-pons (DNP) SI ratio of extremely limited amplitude: 0.0032 ± 0.0216 for gadoterate meglumine and 0.0019 ± 0.0346 for gadobutrol. This increase was shown to be non-significant, as stated in the results section and in Fig. 3: “in neither case (gadoterate meglumine and gadobutrol) was the difference significantly different from 0 (*P* > 0.01)”.

The means of the DNP SI ratios were extremely close to the minimum and far from the maximum of the 95% confidence intervals: mean [min; max] were 0.0032 [0.0025; 0.0495] for gadoterate meglumine and 0.0019 [0.0017; 0.0235] for gadobutrol. Therefore, it is likely that the values within these intervals did not follow a normal Gaussian distribution and that the statistical tests used for comparisons were not appropriate.

Furthermore, the authors compared their results with those of Radbruch et al. [2] and Weberling et al. [3]. They concluded that “linear regression revealed overall similarity between our findings and those of other authors regarding macrocyclic GBCAs”. The publications referenced for comparison found no significant effect on the DNP SI ratio with macrocyclic GBCAs, even following multiple injections.

Contrary to these previous publications, the authors mentioned a visible T1-weighted hyperintensity in approximately one third of the patients. No specific analyses were performed to correlate hyperintensities and numbers of injections. Interestingly, the example shown in Fig. 4a, b is from a patient who received the highest level of injections, and the signal was already visible in the DN prior to injection of gadoterate meglumine.

We would also like to highlight several inaccuracies or mistakes along the publication such as the use of gadoteridol name instead of gadoterate meglumine in the results section, the nonscientific representation of the Fig. 3 which shows the means of SI ratios without error bars and whose scale should be extended by a factor 10 to include such error bars, and finally the inconsistency regarding the maximum number of gadobutrol injections in Table 1 compared to the results Sections (14 vs. 15).

To conclude, we consider that Splendiani et al. should have been cautious and consistent in their conclusions, as they were not fully supported by their results. The data reported in this article confirm the previous studies, showing no evidence of gadolinium retention in brain after multiple injections of the macrocyclic GBCAs.

## References

[1] Splendiani A, Perri M, Marsecano C, Vellucci V, Michelini G, Barile A, Di Cesare E (2017) Effects of serial macrocyclic-based contrast materials gadoterate meglumine and gadobutrol administrations on gadolinium-related dentate nuclei signal increases in unenhanced T1-weighted brain: a retrospective study in 158 multiple sclerosis (MS) patients. Radiol Med. 10.1007/s11547-017-0816-9**(Epub ahead of print)** 10.1007/s11547-017-0816-9 28952018

[2] Radbruch A, Weberling LD, Kieslich PJ (2015). Gadolinium retention in the dentate nucleus and globus pallidus is dependent on the class of contrast agent. Radiology.

[3] Weberling LD, Kieslich PJ, Kickingereder P (2015). Increased signal intensity in the dentate nucleus on unenhanced T1-weighted images after gadobenate dimeglumine administration. Invest Radiol.

